# Alterations in Multidimensional Functional Connectivity Architecture in Preschool Children with Autism Spectrum Disorder

**DOI:** 10.3390/brainsci16010091

**Published:** 2026-01-15

**Authors:** Jiannan Kang, Xiangyu Zhang, Zongbing Xiao, Zhiyuan Fan, Xiaoli Li, Tianyi Zhou, He Chen

**Affiliations:** 1 College of Electronic & Information Engineering, Hebei University, Baoding 071002, China; kangjiannan81@163.com (J.K.); 13633195227@163.com (X.Z.); zongbingxiao@163.com (Z.X.); 13613598764@163.com (Z.F.); 2 State Key Laboratory of Cognitive Neuroscience and Learning, Beijing Normal University, Beijing 100875, China; xiaoli@bnu.edu.cn; 3 Department of Psychology, Faculty of Arts and Sciences, Beijing Normal University at Zhuhai, Zhuhai 519088, China; 4 Beijing Key Laboratory of Applied Experimental Psychology, National Demonstration Center for Experimental Psychology Education, Faculty of Psychology, Beijing Normal University, Beijing 100875, China; 5 School of Automation Science and Engineering, South China University of Technology, Guangzhou 510641, China

**Keywords:** autism spectrum disorder, EEG, graph theory, dynamic brain networks, state entropy, between-frequency functional network

## Abstract

**Background:** Autism Spectrum Disorder (ASD) is a type of neurodevelopmental disorder, and its exact causes are currently unknown. Neuroimaging research suggests that its clinical features are closely linked to alterations in brain functional network connectivity, yet the specific patterns and mechanisms underlying these abnormalities require further clarification. **Methods:** We recruited 36 children with ASD and 36 age- and sex-matched typically developing (TD) controls. Resting-state EEG data were used to construct static and dynamic low- and high-order functional networks across four frequency bands (δ, θ, α, β). Graph-theoretical metrics (clustering coefficient, characteristic path length, global efficiency, local efficiency) and state entropy were applied to characterize network topology and dynamic transitions between integration and segregation. Additionally, between-frequency networks were built for six band pairs (δ-θ, δ-α, δ-β, θ-α, θ-β, α-β), and network global measures quantified cross-frequency interactions. **Results:** Low-order networks in ASD showed increased δ and β connectivity but decreased θ and α connectivity. High-order networks demonstrated increased δ connectivity, reduced α connectivity, and mixed alterations in θ and β. Graph-theoretical analysis revealed pronounced α-band topological disruptions in ASD, reflected by a lower clustering coefficient and efficiency and higher characteristic path length in both low- and high-order networks. Dynamic analysis showed no significant entropy changes in low-order networks, while high-order networks exhibited time- and frequency-specific abnormalities, particularly in δ and α (0.5 s window) and δ (6 s window). Between-frequency analysis showed enhanced β-related coupling in low-order networks but widespread reductions across all band pairs in high-order networks. **Conclusions:** Young children with ASD exhibit coexisting hypo- and hyper-connectivity, disrupted network topology, and abnormal temporal dynamics. Integrating hierarchical, dynamic, and cross-frequency analyses offers new insights into ASD neurophysiology and potential biomarkers.

## 1. Introduction

As a complex neurodevelopmental disorder, autism spectrum disorder (ASD) is characterized by two core features: persistent difficulties in social communication and interaction, and restricted and repetitive patterns of behavior, interests, or activities [1,2]. In recent years, the global prevalence of autism has shown a significant upward trend. According to data from the U.S. Centers for Disease Control and Prevention, approximately one in every 36 children is diagnosed with ASD, posing a serious challenge to public health systems [3]. The etiology of autism has not yet been fully elucidated, but the current scientific consensus is that it results from interactions between genetic and environmental factors and represents a complex polygenic disorder [4,5].

Brain imaging technologies, represented by electroencephalography (EEG), functional magnetic resonance imaging (FMRI), and positron emission tomography (PET), have now become important research tools for exploring brain function and neurodynamics [6]. EEG is a non-invasive neuroimaging tool that is frequently employed for the diagnosis of various neurological disorders [7,8]. Research has demonstrated that any abnormal activity of neurons leaves distinctive signatures in EEG signals [9]. Therefore, functional connectivity analysis based on EEG may provide an important window for understanding abnormal brain activity in ASD.

Numerous studies have attempted to characterize abnormal functional connectivity in ASD; however, the findings have been inconsistent. Some studies support the “hypoconnectivity” hypothesis [10], suggesting reduced synchronous activity between different brain regions, while others have reported “hyperconnectivity” phenomena [11]. Additionally, some research has found the coexistence of both patterns [12]. This inconsistency suggests that abnormal brain connectivity in ASD may not follow a single model, but rather represents a complex manifestation influenced by multiple factors such as analytical scale, participant age, and methodological differences. Such complexity makes it difficult for analyses based on a single modality or a single scale to capture the full extent of ASD-related abnormalities.

Low-order functional connectivity (LOFC), as a foundational metric in brain network research, characterizes pairwise connections between nodes based on direct correlations between regional brain signals [13,14]. However, this approach is limited in its ability to capture complex high-order interactions between brain regions, such as the coordinated changes among different connectivity patterns, and thus cannot fully reveal the organizational principles of brain networks [15]. High-order functional connectivity (HOFC) is constructed by calculating correlations between column vectors of the LOFC matrix, thereby creating a second-order network that reflects “correlations of correlations.” With this method, the analytical focus shifts from direct temporal synchrony to the associations among spatial connectivity patterns [16,17]. This makes it possible to uncover more abstract and complex organizational rules within networks and may more sensitively detect atypical coordination patterns present in ASD networks.

Nevertheless, both low-order functional connectivity and high-order functional connectivity have traditionally been constructed under static assumptions, overlooking the fact that the brain is inherently a dynamically changing complex system [18,19]. Studies have shown that brain functional connectivity continuously fluctuates across different temporal scales, and this dynamic nature is closely related to the flexibility of cognitive processes [20]. Therefore, relying solely on static network analysis makes it difficult to fully capture the difficulties individuals with ASD in information processing and state transitions. In recent years, dynamic functional network analysis has attracted increasing attention. Commonly using techniques such as the sliding window method, it captures the time-varying characteristics of brain networks and provides new perspectives for understanding the brain’s dynamic balance between integration and segregation states [21,22]. Integration and segregation are two fundamental principles of brain functional organization: integration is responsible for the global coordination of information across brain regions, while segregation focuses on specialized processing within local modules [23,24]. The flexible switch between these two states is crucial for maintaining normal cognitive function. At the same time, graph theory, as an important tool in complex network analysis, has become a core method for exploring the mechanisms of information processing in brain networks and is widely used to quantify the topological properties of brain networks, thereby assessing their integrative and segregative capacities [19,25]. In dynamic analyses, metrics such as state entropy can further characterize the complexity and predictability of network state transitions, reflecting the brain’s capacity for dynamic regulation.

EEG synchronization can be divided into two modes: within-frequency coupling, which occurs within a specific frequency range, and cross-frequency coupling, which occurs between different frequency bands [26]. Given that research has shown that autism spectrum disorder (ASD) is associated with specific brain regions and frequency bands, it is particularly necessary to explore it from the overall perspective of intra- and inter-band interactions, rather than isolated brain regions [27,28]. Previous ASD studies have examined spectral power and functional connectivity [29,30]. Although such connectivity studies consider interactions between regions, they often ignore interactions between frequencies and more complex synchronizations. Cross-frequency coupling is not innate; rather, it develops and becomes refined from early childhood through the school-age stage. It is regarded as a fundamental mechanism enabling information exchange across neural oscillations at different spatial and temporal scales, thus supporting complex cognitive functions such as working memory, language processing, and cognitive control. The preschool period (ages 3–6) is a crucial window in which this key network property undergoes rapid shaping. As a core mechanism for integration and coordination between different brain regions and various information processing rates, cross-frequency coupling disruptions are considered a potential neural basis for the fragmented information processing and difficulty in forming coherent perception and thought seen in individuals with ASD. Therefore, investigating cross-frequency coupling in ASD children during this developmental stage provides a powerful perspective for assessing the maturation of their brain information integration functions. Therefore, analyzing between-frequency connectivity may provide a more comprehensive understanding of ASD. In this study, we employed a multilayer network approach that has been developed to analyze multilayer brain functional connectivity across different frequency bands. Furthermore, we proposed network global metrics to quantify the overall synchronization of functional connectivity in ASD.

In summary, this study has constructed a multidimensional EEG analysis framework. This framework integrates intra- and inter-frequency low- and high-order functional connectivity and static and dynamic network analysis, and introduces a state entropy evaluation method. Its purpose is to systematically reveal the abnormal patterns of brain functional networks in preschool children with ASD.

## 2. Materials and Methods

### 2.1. Participants

This study included a total of 36 children with Autism Spectrum Disorder (ASD) and 36 typically developing (TD) children matched for age and gender. Before the experiment began, all participants signed written informed consent forms, and their guardians were fully informed of the research process and content. This study strictly adheres to the ethical principles of the Declaration of Helsinki and has been approved by the Ethics Committee of Ningbo Rehabilitation Hospital (approval number: 2023007).

The inclusion criteria for ASD children were as follows: (a) a diagnosis of ASD confirmed by a qualified child psychiatrist according to the Diagnostic and Statistical Manual of Mental Disorders, Fifth Edition (DSM-5) [31]; (b) age between 4 and 6 years; and (c) written informed consent obtained from their legal guardians. According to the research requirements, individuals who meet any of the following criteria are not included: (a) having a history of neurological disorders such as epilepsy, brain injury, or neurosurgery; (b) having taken antipsychotic drugs or anticonvulsant drugs before the start of the study or during the experiment. In this study, the ASD children had limited language abilities and decreased self-care skills and were able to understand and follow only simple instructions; therefore, the ASD participants were classified as low-functioning individuals.

The inclusion criteria for TD children were: (a) age between 4 and 6 years with normal intelligence, and (b) written informed consent from their guardians. Some participating TD children underwent intelligence assessments, with IQ scores ranging from 90 to 105, which is within the normal range.

### 2.2. EEG Data Acquisition and Preprocessing

The equipment used for EEG data collection in this study was manufactured by Jiangxi Jielian Medical Equipment Co., Ltd., located in Ganjiang New Area, Nanchang, Jiangxi Province, China. All EEG signals in the eyes-open resting state were recorded using an 8-channel EEG acquisition system. Electrodes were positioned according to the international 10–20 system (F3, F4, T3, C3, C4, T4, O1, and O2), with the Cz site serving as the reference electrode. During data collection, the sampling rate was set to 1000 Hz, and electrode impedances were kept below 20 kΩ. Under the guidance of technicians, each child completed approximately five minutes of EEG recording in a relaxed seated position.

**Figure 1:**
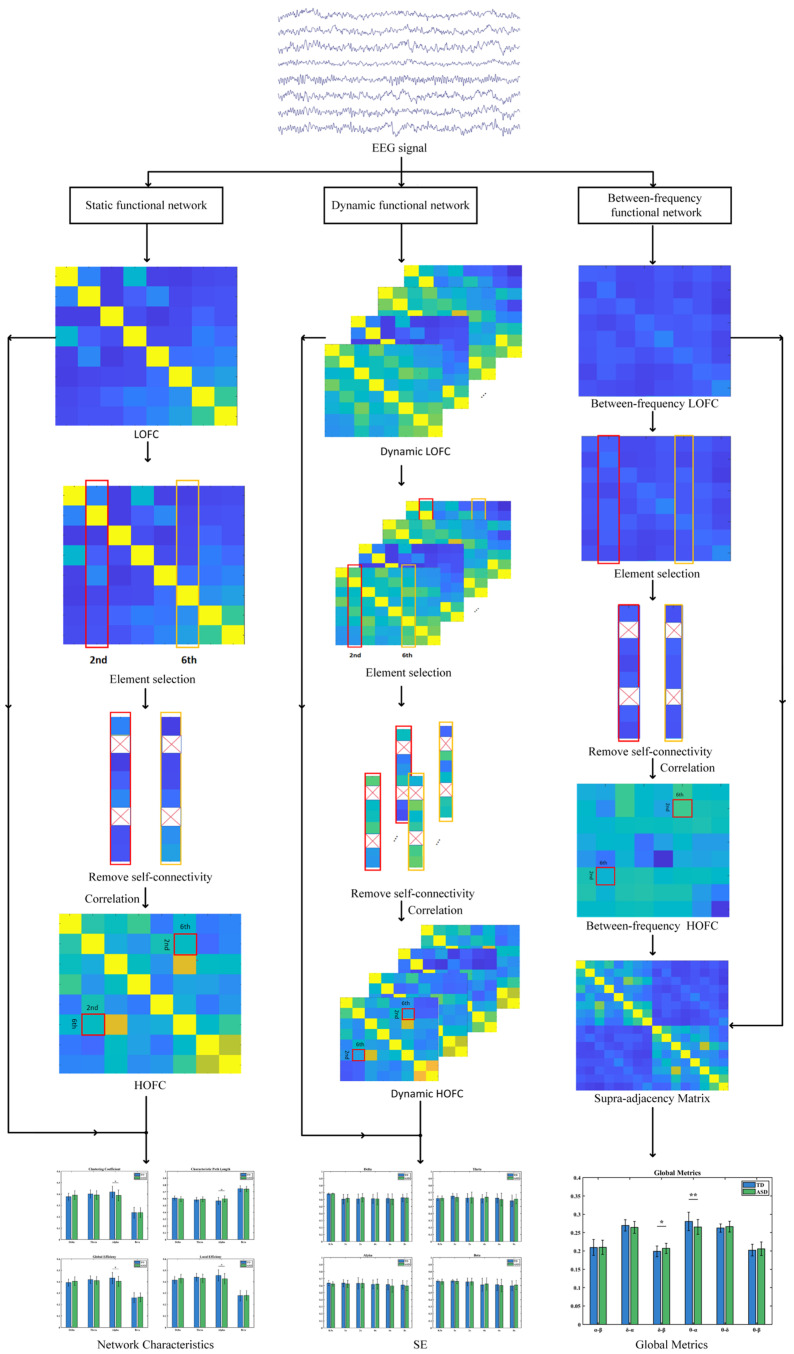
The overall experimental design and analysis process (* Indicates *p* < 0.05, ** Indicates 0.001 < *p* < 0.01).

The collected EEG signals underwent offline preprocessing in the MATLAB R2024a environment using the EEGLAB toolbox (v2024.2). To improve data quality, the raw signals were processed sequentially as follows: Firstly, a 50 Hz notch filter was applied to eliminate power line interference; Next, a 0.5–45 Hz band-pass filter was used to retain valid components of neural oscillations. To effectively suppress signal artifacts caused by eye movements, blinks, and electromyographic (EMG) activity, this study employed a denoising strategy that combines ensemble empirical mode decomposition (EEMD) with independent component analysis (ICA). This combined approach has been proven to effectively filter out common artifacts and ensure the reliability of subsequent analyses. Afterward, manual screening was conducted to directly identify and remove any remaining noisy segments. Finally, all data were re-referenced to a common baseline, and feature extraction and analyses were performed based on the preprocessed signals. The overall experimental analysis framework is illustrated in Figure 1.

### 2.3. Construction of Low-Order and High-Order Brain Functional Networks

In this study, we constructed functional brain networks in four frequency bands—delta (1–4 Hz), theta (4–8 Hz), alpha (8–13 Hz), and beta (13–30 Hz)—for both TD and ASD children. Phase-locking value (PLV) was used as a measure of LOFC to investigate the phase synchronization mechanisms of neural oscillations. After calculating the electroencephalogram (EEG) phase of each channel, further analyze the phase lag between every two channels, and construct an 8 × 8 adjacency matrix based on the obtained phase lag values (PLVs). In this matrix, a higher PLV reflects stronger synchrony between the corresponding channels [32,33].

Specifically, to estimate the instantaneous phases $\varphi_{u}(t)$ and $\varphi_{v}(t)$ of the EEG signals *u*(*t*) and *v*(*t*), we used the Hilbert transform (HT) to construct the analytic signal *H*(*t*), which can be expressed as:

(1) $$H_{u}(t)=u(t)+iHT_{u}(t)$$

(2) $$H_{v}(t)=v(t)+iHT_{v}(t)$$

where ${\mathrm{HT}}_{u}(t)$ and${\;\mathrm{HT}}_{v}(t)$ represented the corresponding HT, which can be expressed as:

(3) $$HT_{u}(t)=\frac{1}{\pi }P\cdot V\cdot \int_{-\infty }^{\infty }\frac{u(t^{\prime })}{t-t^{\prime }}dt^{\prime }$$

(4) $$HT_{v}(t)=\frac{1}{\pi }P\cdot V\cdot \int_{-\infty }^{\infty }\frac{v(t^{\prime })}{t-t^{\prime }}dt^{\prime }$$

where P·V denoted the Cauchy principal value. The phases of the analytic signals${\;\varphi }_{u}(t)$ and $\varphi_{v}(t)$ were then calculated as follows:

(5) $$\varphi_{u}(t)=\arctan\frac{HT_{u}(t)}{u(t)}$$

(6) $$\varphi_{v}(t)=\arctan\frac{HT_{v}(t)}{v(t)}$$

Subsequently, the calculation of the PLV was given by:

(7) $$PLV=\left|\frac{1}{N}\sum_{j=0}^{N-1}e^{i(\varphi_{u}(j\Delta t)-\varphi_{v}(j\Delta t))}\right|$$

where *j* represented the *j*-th sampling point, and *N* was the total number of samples for each signal; t denoted the time point and ∆*t* was the sampling period. The PLV reflected the phase synchrony strength between paired nodes.

The construction process of the LOFC matrix was as follows: First, the preprocessed EEG signals were subjected to Hilbert transform to extract the instantaneous phase sequences for each channel. Subsequently, by calculating the PLV between all pairs of channels in the network, a complete LOFC matrix was generated.

The construction of the HOFC matrix is based on the LOFC matrix. The specific process is as follows: First, select any two columns from the obtained LOFC matrix, and remove the autocorrelation terms before calculating the inter-column correlation. Specifically, when calculating the correlation between the *i*-th and *j*-th columns, the *i*-th and *j*-th elements in these two columns need to be removed, respectively. Afterwards, perform Fisher’s z-transformation on each column, and finally obtain the HOFC matrix by calculating the Pearson correlation coefficient between any two columns in the transformed matrix.

In this study, we employed a method based on low-order functional connectivity matrix column correlation to construct high-order networks. Specifically, high-order networks are obtained by calculating the Pearson correlation between column vectors representing the connectivity patterns of various brain regions in the adjacency matrix of low-order networks. In this high-order network, nodes are consistent with the low-order network and still represent the original brain regions; but what the connecting edge represents is no longer the direct phase synchronization between signals, but the similarity of functional connectivity patterns between two brain regions across the entire brain. This method aims to capture more global brain network collaborative configuration relationships beyond paired interactions.

### 2.4. Network Characteristics of Low- and High-Order Brain Functional Networks

To characterize the topological organization of brain functional networks, we employed graph theory to calculate four core topological metrics for static low-order and high-order brain functional networks: clustering coefficient (CC), characteristic path length (CPL), global efficiency (GE), and local efficiency (LE) [19,25]. For the calculation of network characteristics, weighted brain functional networks were constructed based on the original PLV, and graph theoretical formulas suitable for weighted networks were applied. This approach aimed to preserve the continuous weight information of the network and avoid potential bias caused by arbitrarily set thresholds.

CC: This metric quantifies the degree of dense local interconnectivity within a network. A higher clustering coefficient usually indicates the presence of specialized information processing modules in the network, suggesting greater local processing efficiency.

(8) $$CC=\frac{1}{n}\sum_{i\in \theta }\frac{\sum_{j,h\in \theta }\left(w_{ij}^{pcc}w_{ih}^{pcc}w_{jh}^{pcc}\right)}{\sum_{j\in \theta }w_{ij}^{pcc}(\sum_{j\in \theta }w_{ij}^{pcc}-1)}$$

CPL: This metric measures the efficiency of global information transmission in the network, and its value is equal to the average of the shortest path lengths between all pairs of nodes. A shorter path length indicates that information needs to undergo fewer intermediary steps when transmitted in the brain, indicating a stronger overall integration ability of the network.

(9) $$CPL=\frac{1}{n}\sum_{i\in \theta }\frac{\sum_{j\in \theta ,j\neq i}d_{ij}}{n-1}$$

GE: This metric is a complementary measure to characteristic path length and directly evaluates the efficiency of parallel information transfer at the global network level. Higher values indicate a stronger ability of the network as a whole to exchange and integrate information.

(10) $$GE=\frac{1}{n}\sum_{i\in \theta }\frac{\sum_{j\in \theta ,j\neq i}{\left(d_{ij}\right)}^{-1}}{n-1}$$

LE: This metric measures the local information transmission capacity of the network and the degree of aggregation of neighboring nodes. It is defined as the harmonic mean of the shortest path lengths between all pairs of nodes within the same subnetwork. A higher value indicates stronger information processing efficiency within the subnetwork.

(11) $$LE=\frac{1}{n}\sum_{i\in \theta }\frac{\sum_{j,h\in \theta ,j\neq i}{\left(w_{ij}^{pcc}w_{ij}^{pcc}\left[d_{jh}{\left(\theta_{i}\right)}^{-1}\right]\right)}^{\frac{1}{3}}}{\sum_{j\in \theta }w_{ij}^{pcc}(\sum_{j\in \theta }w_{ij}^{pcc}-1)}$$

### 2.5. Construction of Dynamic Low- and High-Order Brain Functional Networks

This study focuses on the analysis of four typical EEG frequency bands: delta band, theta band, alpha band, and beta band, and for each frequency band, six different time window lengths (0.5 s, 1 s, 2 s, 4 s, 6 s, and 8 s) were set to comprehensively investigate the dynamic characteristics of brain functional networks across multiple time scales. The selection of window lengths was based on protocols verified as effective in dynamic brain network studies of amnestic mild cognitive impairment [18]. This range of window lengths was intended to capture both sub-second rapid regulation and slower oscillations over several seconds, thereby ensuring a thorough assessment of the dynamic integration and segregation capacities of ASD children across different time scales.

The construction process for dynamic low-order brain functional networks was as follows: First, the EEG signals in the delta, theta, alpha, and beta frequency bands were processed separately, and the signals were segmented using the six window lengths (0.5 s, 1 s, 2 s, 4 s, 6 s, and 8 s), with a window overlap rate of 20%. This setting not only effectively prevented the loss of key dynamic information, but also significantly improved temporal resolution, enabling the continuous and stable capture of rapid network state transitions, while effectively suppressing spectral leakage and edge effects caused by window segmentation [33,34]. Subsequently, the signals within each window were processed according to a unified procedure: instantaneous phase sequences for each channel were extracted via Hilbert transform, and PLV were calculated between all channels to form an LOFC matrix for each time window. Through this procedure applied to all EEG time segments, a complete series of LOFC matrices was generated, thereby constituting the dynamic low-order brain functional network.

The construction process for dynamic high-order brain functional networks was as follows: Based on the above dynamic low-order network results, dynamic high-order networks were further constructed. For each LOFC matrix, the following procedure was performed: any two columns of the matrix were selected, and self-connection elements were removed prior to correlation calculation. Specifically, when computing the correlation between the *i*-th and *j*-th columns, the *i*-th and *j*-th elements were removed from the respective columns. After removing self-connections, each column was processed through Fisher’s z-transformation, and then the Pearson correlation coefficient between any two columns of the transformed matrix was calculated to obtain the corresponding HOFC matrix. By performing this process on all EEG time segments, a complete series of HOFC matrices was generated, thereby constituting the dynamic high-order brain functional network.

### 2.6. State Entropy of Dynamic Brain Functional Networks

To characterize dynamic state transitions in brain networks, this study utilized the time-varying network participation coefficient and applied the K-means clustering algorithm to define network states [35]. Specifically, the number of clusters was set to 2, distinguishing between two typical states in the network: integration and segregation. The integration state reflects dense intermodular connections and enhanced interactions, while the segregation state reflects sparse intermodular connections and relatively independent functioning among modules. To improve the stability of clustering and reduce sensitivity to initial values, the clustering process was repeated 50 times.

To quantify the complexity of transitions between integrated and segregated states in dynamic brain networks, this study introduces the State Entropy (SE) as an indicator. In specific calculations, integrated and segregated states are encoded as “1” and “0”, respectively, resulting in four possible state transition sequences (00, 01, 10, 11). State entropy is obtained by calculating the entropy value of the probabilities of each sequence, and the calculation formula is as follows.

(12) $$SE=-\frac{1}{\log(M)}\sum_{i=1}^{M}p_{i}\log(p_{i})$$

### 2.7. Construction of Low-Order and High-Order Brain Functional Networks Between Frequency Bands

To further investigate the interactions between different neural oscillatory rhythms in the brain, this study introduced between-frequency functional connectivity analysis in addition to within-frequency functional connectivity analysis. We calculated six representative between-frequency PLV pairs: α-β, α-δ, θ-δ, δ-β, β-θ, and α-θ.

The construction process for between-frequency LOFC matrices was as follows: For each between-frequency pair (using α-β as an example), the EEG signals from the same channel were filtered into both the α and β frequency bands. The Hilbert transform was then applied to the signals in each band separately to extract their instantaneous phase time series. Finally, the PLV between the instantaneous phase series from the two different frequency bands was calculated, serving as an index of the between-frequency coupling strength of neural activity in that channel between the α and β bands. This process was applied in parallel to all predefined channel pairs, resulting in a functional connectivity matrix for each between-frequency combination.

The construction of the between-frequency higher-order functional connectivity (HOFC) matrix is based on the between-frequency lower-order functional connectivity (LOFC) matrix obtained in the previous step. The specific steps are as follows: Select any two columns from the LOFC matrix. Before calculating the inter-column correlation, the self-connection terms in these two columns need to be removed (when calculating the correlation between the *i*-th and *j*-th columns, the *i*-th and *j*-th elements in each column need to be removed, respectively). Subsequently, perform Fisher’s z-transformation on each processed column. Finally, by calculating the Pearson correlation coefficient between any two columns in the transformed matrix, the between-frequency HOFC matrix is obtained.

After constructing the four within-frequency and six between-frequency connectivity matrices, we further integrated the multi-frequency interaction information across the whole brain by introducing a supra-adjacency matrix for higher-order analysis. This matrix is a symmetric 16 × 16 structure, defined as follows: the diagonal blocks consist of the within-frequency 8-channel PLV connectivity matrices, representing phase synchronization patterns within each frequency band. The off-diagonal blocks are populated with the six between-frequency PLV connectivity matrices, reflecting between-frequency coupling between different neural oscillatory rhythms.

To extract a metric quantifying cooperative interactions between frequency bands from this complex network, we computed the arithmetic mean of all elements within the off-diagonal blocks of the supra-adjacency matrix. This network global metric reflects the overall strength of functional connectivity between different frequency bands—the higher its value, the stronger the brain’s between-frequency coordination and information integration across neural oscillatory scales.

### 2.8. Statistical Analysis

The statistical analysis in this study was conducted on the MATLAB platform. To examine the inter-group differences in static and dynamic brain functional connectivity (including low-order and high-order), network topological properties, dynamic network state transitions, and between-frequency connectivity between children with autism spectrum disorder (ASD) and typically developing (TD) children, we employed independent sample *t*-tests for inter-group comparisons. Additionally, to control for false positives that may arise from multiple comparisons, all *p*-values obtained from *t*-tests were corrected using the False Discovery Rate (FDR).

## 3. Results

In this study, we adopted a multidimensional framework combining static, dynamic, and between-frequency analyses to systematically investigate differences in brain functional networks between ASD and TD children. Specifically, the analyses encompassed three levels: first, within each of the four frequency bands, the connectivity strength and graph-theoretical topological characteristics of both low-order and high-order functional networks were assessed; second, To reveal the temporal dynamic characteristics of network transitions between integrated and separated states, this study employed six time windows of varying durations to assess the state entropy of dynamic low-order and high-order networks, respectively. Finally, by calculating six sets of between-frequency functional connectivity and constructing a supra-adjacency matrix, a network global metric was computed to quantify the overall level of coordination across different neural oscillatory rhythms in the brain.

### 3.1. Low-Order Brain Functional Network Connectivity Results

#### 3.1.1. LOFC Network Connection Strength

**Figure 2:**
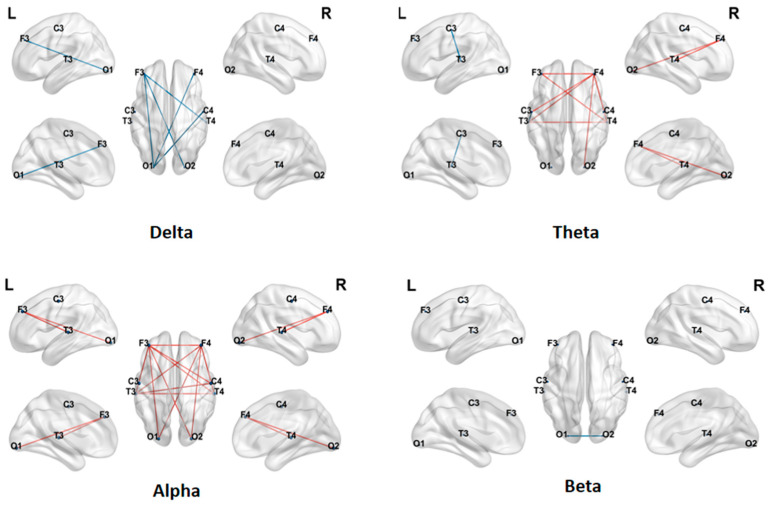
LOFC differences between TD and ASD children in four frequency bands. Red line: TD > ASD, Blue line: TD < ASD.

The analysis results comparing LOFC strength across four frequency bands between ASD and TD children are shown in Figure 2. The study found significant differences in connectivity patterns across different frequency bands: In the delta band, the ASD group exhibited widespread hyperconnectivity, with significantly greater connectivity strength than the TD group; in the theta band, a mixed pattern of both increased and decreased connectivity was observed, but most connections still showed significantly higher strength in the TD group; in the alpha band, the connectivity strength in the ASD group was generally lower than that in the TD group, displaying a clear pattern of hypoconnectivity; and in the beta band, only a small number of connections showed significant group differences, with some connections exhibiting relatively higher strength in the ASD group. Overall, alterations in brain functional connectivity among children with ASD demonstrated marked frequency-band dependence.

#### 3.1.2. Network Characteristics of LOFC Network

**Figure 3:**
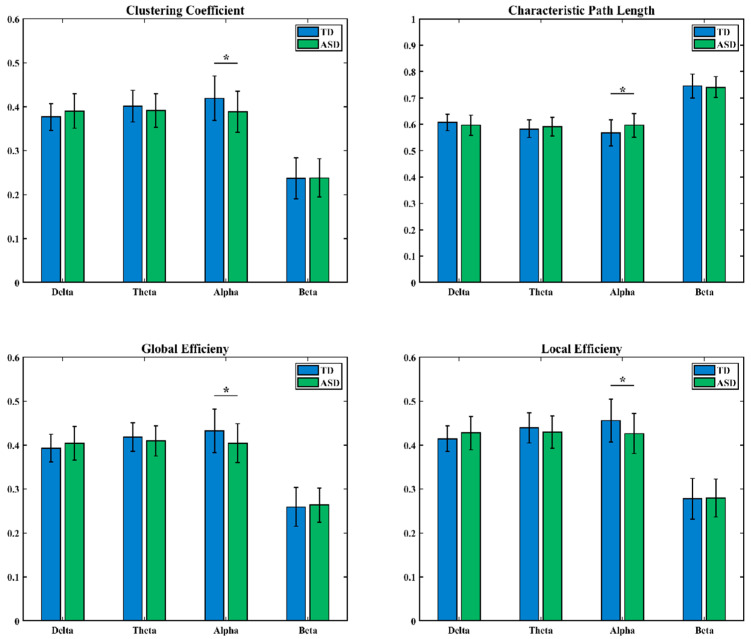
Differences in LOFC network characteristics between TD children and ASD children (*: 0.01 < *p* < 0.05).

Graph theory analysis was conducted on the LOFC networks of ASD and TD children in the delta, theta, alpha, and beta frequency bands, focusing on four topological properties: clustering coefficient (CC), characteristic path length (CPL), global efficiency (GE), and local efficiency (LE). The distributions of these metrics for both groups are shown in Figure 3, with blue representing the TD group and green representing the ASD group.

In the alpha frequency band, statistical results showed significant differences in CC, CPL, GE, and LE between the two groups of subjects (* indicates 0.01 < *p* < 0.05).

### 3.2. High-Order Brain Functional Network Connectivity Results

#### 3.2.1. HOFC Network Connection Strength

**Figure 4:**
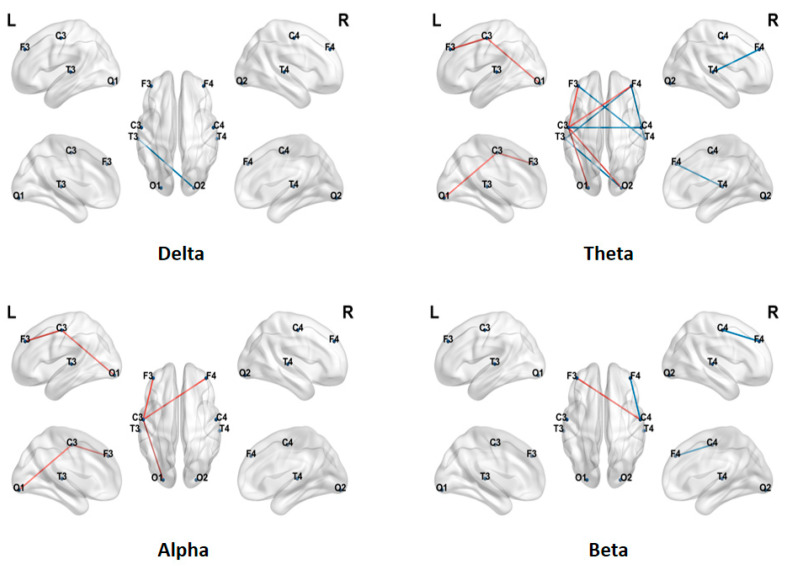
HOFC differences between TD and ASD children in four frequency bands. Red line: TD > ASD, Blue line: TD < ASD.

Further comparison of HOFC strength across four frequency bands between ASD and TD children is shown in Figure 4. Overall, the two groups exhibited a frequency-specific pattern in HOFC similar to that observed for LOFC. Detailed statistical analysis revealed the following: In the delta frequency band, the functional connectivity strength in children with Autism Spectrum Disorder (ASD) is significantly higher than that in typically developing (TD) children; the connectivity pattern in the θ frequency band exhibits a mixed increase and decrease in the ASD group, whereas in the α frequency band, the connectivity strength in the TD group is significantly higher than that in the ASD group; and in the beta band, a similar trend to the theta band was observed, with some connections in the ASD group showing increased strength and others decreased strength, resulting in a non-uniform distribution of connectivity.

#### 3.2.2. Network Characteristics of HOFC Network

**Figure 5:**
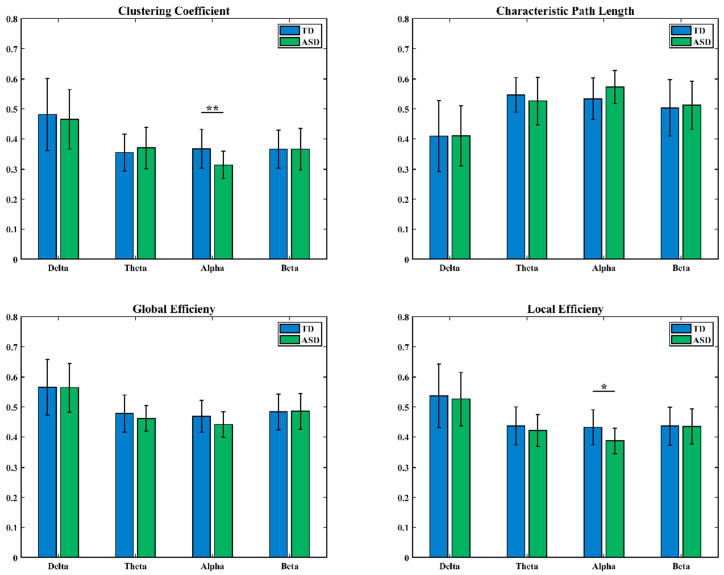
Differences in HOFC network characteristics between TD children and ASD children (*: 0.01 < *p* < 0.05, **: 0.001 < *p* < 0.01).

In this study, we calculated the CC, CPL, GE, and LE of the HOFC networks in the delta, theta, alpha, and beta bands. Figure 5 presents box plots for these indicators (blue: typical development group TD; green: autism spectrum disorder group ASD). The results indicate that the GE and LE of the TD group are higher than those of the ASD group in the theta and alpha frequency bands; in the alpha frequency band, the CPL of the ASD group is higher. Statistical tests further reveal significant differences in CC and LE between the two groups in the alpha frequency band (* indicates 0.01 < *p* < 0.05, ** indicates 0.001 < *p* < 0.01).

### 3.3. The Difference in Dynamic State Transitions Between Low-Order and High-Order Networks

In this study, state entropy was used to quantify the dynamic transitions of brain functional networks between integration and segregation states, and to compare group differences between ASD and TD children. Considering that the stability of dynamic connectivity is influenced by window length—with more pronounced metastable fluctuations observed in shorter windows (≤1 s)—we systematically analyzed the state entropy of both low-order and high-order networks across multiple window scales.

#### 3.3.1. Dynamic Low-Order Brain Functional Network State Entropy

**Figure 6:**
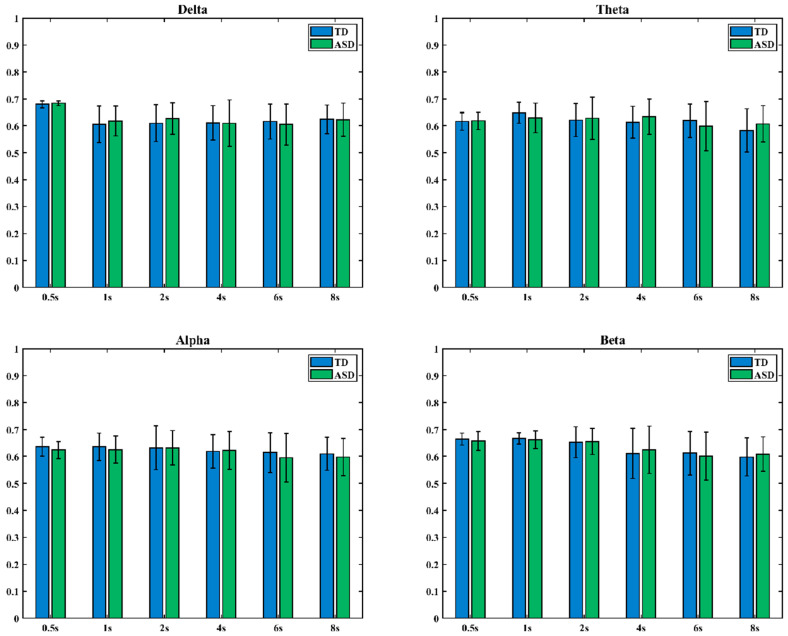
State entropy differences in dynamic low-order brain networks.

Figure 6 presents the comparison of state entropy in dynamic low-order functional networks between ASD and TD children across four frequency bands. The *x*-axis represents window length, and the *y*-axis denotes the state entropy value (TD group: blue; ASD group: green). Statistical analyses showed that there were no significant differences in state entropy between the two groups in any frequency band, either at short time scales (window length ≤ 1 s) or at longer time scales.

#### 3.3.2. Dynamic High-Order Brain Functional Network State Entropy

**Figure 7:**
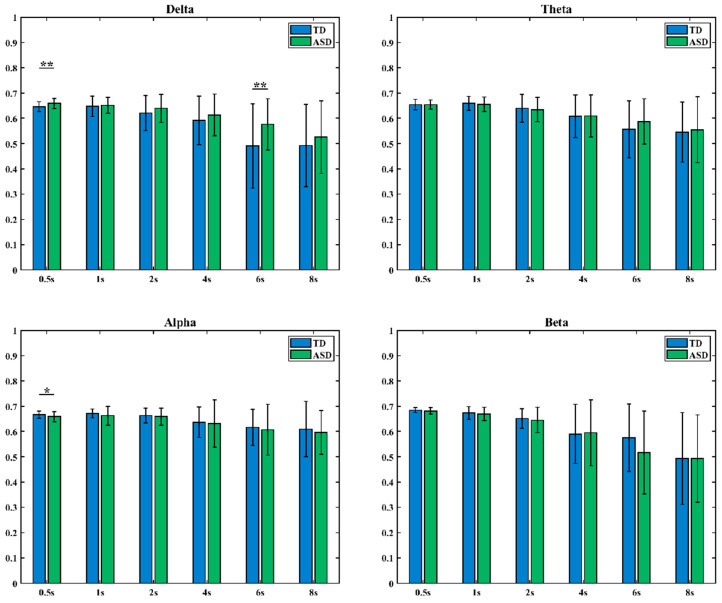
State entropy differences in dynamic high-order brain networks (* Indicates *p* < 0.05, ** Indicates 0.001 < *p* < 0.01).

Figure 7 illustrates the differences in dynamic HOFC network state entropy between children with ASD and TD children across four frequency bands. In the figure, the *x*-axis represents window length, and the *y*-axis represents the state entropy value (blue indicates the TD group, green indicates the ASD group).

The results showed that in the delta band, when the time window was set to 0.5 s and 6 s, there were significant differences in state entropy values between TD and ASD children. In the alpha band, significant differences in state entropy values between the two groups were observed when the time window was set to 0.5 s (* indicates 0.01 < *p* < 0.05, ** indicates 0.001 < *p* < 0.01).

The analysis showed that the state entropy of the ASD group did not exhibit a consistently higher or lower pattern relative to the TD group across different time scales, suggesting the possibility of network global abnormalities in state transitions. Moreover, significant group differences in dynamic high-order networks were distributed inconsistently across frequency bands and time scales, indicating that the mechanisms of state transitions in high-order networks differ from those in low-order networks. Together, these findings support the conclusion that functional integration and segregation of brain networks are impaired in ASD.

### 3.4. Low-Order Brain Functional Network Connectivity Results Between Frequency Bands

#### 3.4.1. Low-Order Brain Functional Network Connectivity Strength Between Frequency Bands

**Figure 8:**
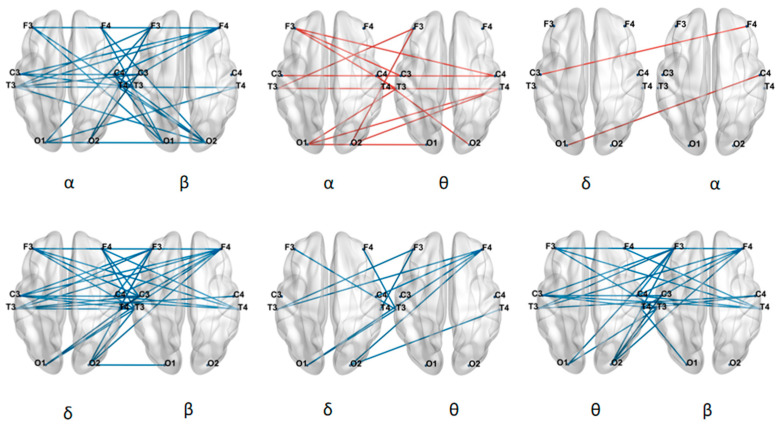
Differences in LOFC of brain networks between frequency bands. Red line: TD > ASD, Blue line: TD < ASD.

We analyzed the LOFC strength of six types of cross-frequency connections between ASD and TD children, as shown in Figure 8. The results revealed that ASD children exhibited significantly higher between-frequency connectivity strength between the β band and the other three bands (δ, θ, and α), as well as between the δ and θ bands, compared to TD children. In contrast, ASD children showed significantly lower between-frequency connectivity strength between the α and θ bands and between the α and δ bands than TD children.

#### 3.4.2. Global Metrics of Low-Order Brain Functional Networks Between Frequency Bands

**Figure 9:**
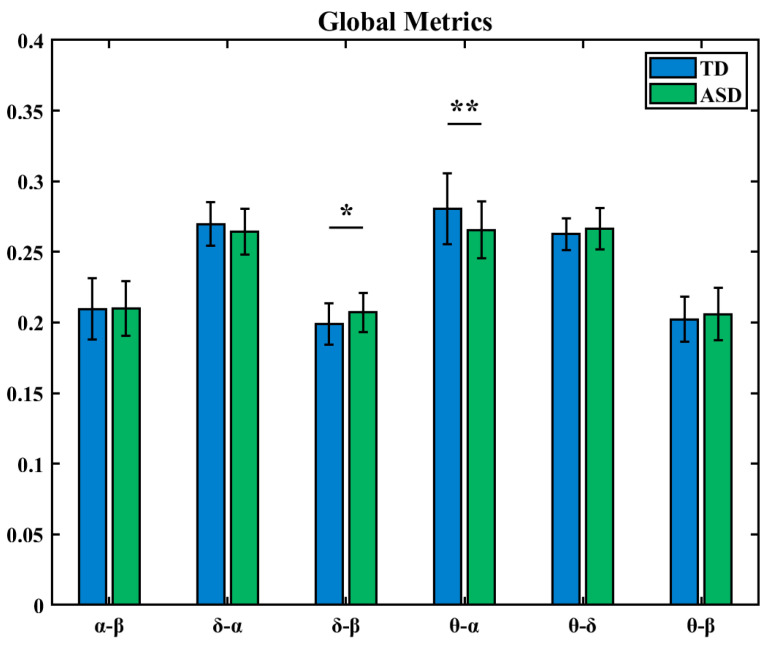
Global metric differences of low-order brain networks between frequency bands (*: 0.01 < *p* < 0.05, **: 0.001 < *p* < 0.01).

Based on the between-frequency LOFC matrices, this study further constructed a network global metric to quantify the overall level of coordination between different neural oscillatory rhythms in the brain. The analysis revealed that TD children exhibited higher global coordination levels than ASD children in the δ-α and θ-α frequency band combinations, while in the α-β, δ-β, β-θ, and δ-θ combinations, their coordination levels were lower than those of ASD children. As shown in Figure 9, Statistical tests further indicated significant differences between the two groups in the coordination patterns of the δ-β and δ-α frequency band pairs (* indicates 0.01 < *p* < 0.05, ** indicates 0.001 < *p* < 0.01).

### 3.5. High-Order Brain Functional Network Connectivity Results Between Frequency Bands

#### 3.5.1. High-Order Brain Functional Network Connectivity Strength Between Frequency Bands

**Figure 10:**
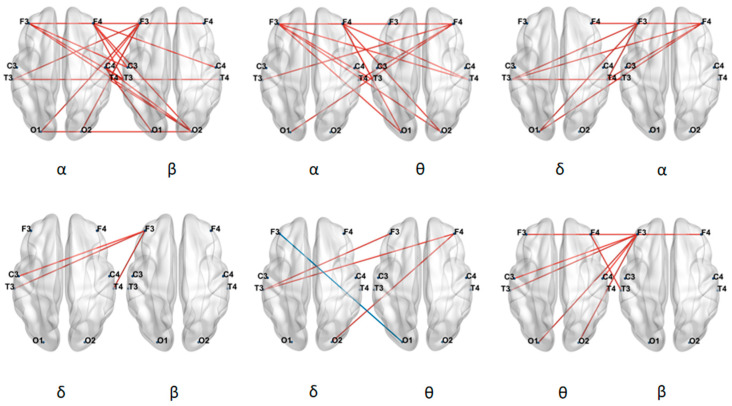
Differences in HOFC of brain networks between frequency bands. Red line: TD > ASD, Blue line: TD < ASD.

We analyzed the HOFC strength of six types of between-frequency connections between ASD and TD children, as shown in Figure 10. The results revealed that ASD children exhibited significantly lower connectivity strength than TD children across all six between-frequency connections.

These findings indicated that between-frequency LOFC in ASD children differs from their between-frequency HOFC. For both between-frequency low-order and high-order connectivity, the TD group showed higher connectivity values than the ASD group in the δ-α and α-θ band pairs. Moreover, the connectivity strength in all between-frequency high-order connections was significantly lower in ASD children compared to TD children, suggesting that both the low-order and high-order between-frequency connectivity patterns are altered in ASD children.

#### 3.5.2. Global Metrics of High-Order Brain Functional Networks Between Frequency Bands

**Figure 11:**
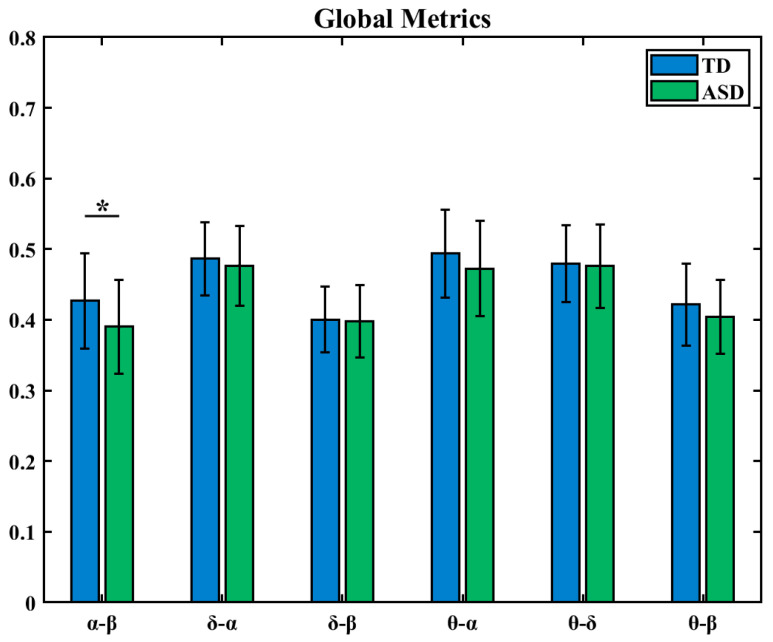
Global metrics differences of high-order brain networks between frequency bands (*: 0.01 < *p* < 0.05).

Meanwhile, we applied global metrics to between-frequency HOFC. The results showed that for all six between-frequency connections, ASD children had lower network global metric values than TD children. As shown in Figure 11, statistical analysis revealed that there was a significant difference in the α-β group (* indicates 0.01 < *p* < 0.05).

## 4. Discussion

By integrating static, dynamic, and between-frequency analyses, this study systematically investigated the abnormal connectivity patterns of brain functional networks in preschool children (aged 3 to 6 years) with ASD. The main findings are as follows:

First, at the level of static connectivity, ASD children exhibited distinct frequency- and hierarchy-dependent abnormalities. In LOFC, the ASD group showed low connectivity across sensor distributions in the theta and alpha bands, indicating a long-term functional integration deficit in brain networks that may impair information transmission and processing efficiency. In contrast, hyperconnectivity was observed in the delta band, which may reflect developmental abnormalities in low-frequency neural rhythms or the formation of compensatory mechanisms. HOFC analysis revealed more complex patterns: ASD children displayed a mixed distribution of increased and decreased connectivity in the theta and beta bands, consistently lower high-order connectivity in the alpha band compared to TD children, and significantly higher connectivity in the delta band. These results suggest that abnormalities in ASD are not limited to direct synchrony but also involve disrupted organization of large-scale brain coordination patterns [36,37]. The differences between low-order and high-order connectivity patterns may reflect multi-scale functional deficits in hierarchical information processing in the ASD brain, offering new perspectives for understanding its heterogeneous neural mechanisms.

Second, in low-order brain functional networks, network characteristics indicated that ASD children exhibited widespread decreases in CC, GE, and LE, as well as increased CPL in key cognitive frequency bands such as alpha. In high-order brain functional networks, network characteristics showed that TD children demonstrated overall topological advantages in the alpha band. For both LOFC and HOFC network characteristics, ASD children had lower GE and LE than TD children in the alpha band. These results demonstrate that brain networks in ASD children have reduced local specialization and network global information integration efficiency [38]. The alpha rhythm is closely related to attention and inner speech processing [39,40]; from a network organization perspective, these findings support the notion of core cognitive deficits in ASD.

Third, the analysis of dynamic brain network state entropy revealed pronounced frequency-specific abnormalities in the brain network dynamics of ASD children. Specifically, increased state entropy in the delta band may indicate instability in the regulation of fundamental neural rhythms, reflected as excessive randomization in network state transitions, whereas decreased state entropy in the alpha band may reflect overly rigid transitions in frequency bands related to higher cognitive functions, with significantly reduced dynamic flexibility. Notably, these dynamic abnormalities were observed only in high-order networks, with no significant differences in low-order networks. This finding suggests that dynamic neural dysfunction in ASD may primarily occur at the level of higher-order network organization coordinating functional module interactions, rather than at the level of basic direct connections. Taken together, these results indicate that network abnormalities in ASD children extend into the temporal domain. Changes in state entropy reveal impaired flexibility in dynamic transitions between integration and segregation states. This abnormal regulation in higher-level network dynamics may be an important neural mechanism leading to reduced cognitive flexibility, diminished adaptability, and stereotyped behaviors in ASD children.

Finally, in the analysis of between-frequency low-order brain functional networks, ASD children showed generally increased connectivity strength between the beta band and other bands (delta, theta, and alpha). In between-frequency high-order networks, ASD children exhibited consistently reduced connectivity strength across all six between-frequency combinations. Network global metrics results further revealed that, in low-order networks, the ASD group showed significantly higher overall coordination in the delta-beta band than the TD group, and significantly lower coordination in the theta–alpha band. In high-order networks, the ASD group exhibited significantly lower coordination in the alpha-beta bands compared to the TD group. In low-order brain networks, ASD children demonstrated higher connectivity and coordination when the beta band was involved. Beta oscillations are commonly associated with alertness and active attention regulation, particularly during cognitive task engagement [41,42]. This excessive activity in the beta band may reflect compensatory mechanisms or abnormal neurodevelopmental trajectories in ASD neural networks. In contrast, high-order networks are responsible for integrating complex interactions between functional systems [43,44]; the widespread reduction in connectivity strength and coordination in ASD children indicates systematic deficits in coordinating oscillatory information across frequency scales. These deficits in higher-level integration capabilities may be closely related to their reduced cognitive flexibility, difficulties in processing complex information, and other core clinical symptoms.

In summary, ASD children demonstrated abnormal connectivity patterns in brain functional networks. Their brains may exhibit instability in basic, local neural synchrony, presenting either increased or decreased connectivity in specific frequency bands. This fundamental instability further impacts higher-order brain functional networks, leading to abnormalities in HOFC and network characteristics, and ultimately affects dynamic regulation across the temporal domain and information integration between frequency bands.

However, this study has certain limitations. First, the sample size was relatively small, and future studies should expand the sample to enhance statistical power. Second, the eight-channel EEG device used has limited spatial resolution; A sparse-node network inherently limits the expressive range of network topology, and metrics such as ‘network global efficiency’ essentially describe the integrative properties of the network formed by the current eight sensors. Therefore, the ‘topological advantages’ or ‘connectivity patterns’ reported in this study should be strictly interpreted as group differences observed within a large-scale subnetwork composed of representative electrodes in the frontal, temporal, central, and occipital regions. These findings provide reliable preliminary evidence for verifying corresponding network integration abnormalities in imaging modalities with higher spatial resolution; future research could incorporate high-density EEG or functional magnetic resonance imaging to validate findings at a finer spatial scale. Additionally, this study primarily focused on network characteristics themselves; future work could combine these abnormal neural metrics with individual cognitive-behavioral data (such as language ability, executive function, and social scores) to further clarify the intrinsic relationships between network abnormalities and clinical manifestations.

Although this study revealed a series of abnormal patterns in the brain functional network of ASD children through a multidimensional framework, there are still some methodological limitations that need to be considered. This study mainly relies on statistical comparisons of network indicators between groups, without directly testing whether the observed functional connectivity patterns themselves differ significantly from the random process using benchmark models (such as phase randomization-based substitute data). This means that our conclusion should reveal systematic relative differences between the ASD and TD groups. However, the strict matching control group design and inter-group comparison paradigm we adopted were based on a reasonable assumption that these two sets of data were affected by similar measurement noise and common artifacts such as volume conduction. Therefore, the discovered inter-group differences are more likely to reflect real inter-group neurophysiological differences rather than shared artifacts. It is necessary for future research to use phase randomization techniques such as Iterative Amplitude Adjusted Fourier Transform (IAAFT) to generate alternative data and construct a zero distribution of connection strength.

The network analysis in this study is based on weighted connections, focusing on revealing the overall differences in connection strength and topological organization between groups. Looking ahead, in order to more accurately analyze the affected core connectivity architecture in ASD, a more refined thresholding strategy can be adopted. For example, as a data-driven thresholding method based on infiltration [45], its core advantage lies in the ability to automatically identify key points (i.e., infiltration phase transition points) where the global connectivity of the network undergoes sudden changes by simulating the sequential removal process of edges, thereby objectively determining the optimal threshold value. This process does not rely on subjectively preset fixed thresholds or network sparsity, and can effectively filter out weak connections that may be caused by noise while maximizing the preservation of meaningful topological structures. In future work, applying such methods to reanalyze the data from this study will help distinguish which connectivity differences constitute fundamental changes in network organization, potentially providing more specific insights into the neural mechanisms of ASD.

## 5. Conclusions

This study established an integrated framework combining static, dynamic, and between-frequency analyses to systematically examine differences in brain functional networks between ASD and TD children aged 3 to 6 years. We not only applied both static and dynamic low-order and high-order functional connectivity methods, but also, for the first time, introduced between-frequency connectivity analysis into the field of ASD research. The aim was to comprehensively reveal the multidimensional abnormal characteristics of ASD in terms of connectivity strength, network topological characteristics, state transition complexity, and between-frequency coordination patterns.

## Data Availability

The data presented in this study are available on request from the corresponding author due to privacy reasons.
